# A Versatile Star PEG Grafting Method for the Generation of Nonfouling and Nonthrombogenic Surfaces

**DOI:** 10.1155/2013/962376

**Published:** 2012-12-20

**Authors:** Pradeep Kumar Thalla, Angel Contreras-García, Hicham Fadlallah, Jérémie Barrette, Gregory De Crescenzo, Yahye Merhi, Sophie Lerouge

**Affiliations:** ^1^Laboratory of Endovascular Biomaterials (LBeV), Research Centre, Centre Hospitalier de l'Université de Montreal (CRCHUM), 2099 Alexandre de Sève, Montreal, QC, Canada H2L 2W5; ^2^Department of Mechanical Engineering, École de Technologie Supérieure (ÉTS), 1100 Boulevard Notre-Dame Ouest, Montreal, QC, Canada H3C 1K3; ^3^Department of Engineering Physics, École Polytechnique de Montreal, P.O. Box 6079, Succ. Centre-Ville, Montreal, QC, Canada H3C 3A7; ^4^Laboratory of Thrombosis and Haemostasis Research Centre, Montreal Heart Institute, 5000 Belanger Street, Montreal, QC, Canada H1T 1C8; ^5^Department of Chemical Engineering, École Polytechnique de Montreal, P.O. Box 6079, Succ. Centre-Ville, Montreal, QC, Canada H3C 3A7

## Abstract

Polyethylene glycol (PEG) grafting has a great potential to create nonfouling and nonthrombogenic surfaces, but present techniques lack versatility and stability. The present work aimed to develop a versatile PEG grafting method applicable to most biomaterial surfaces, by taking advantage of novel primary amine-rich plasma-polymerized coatings. Star-shaped PEG covalent binding was studied using static contact angle, X-ray photoelectron spectroscopy (XPS), and quartz crystal microbalance with dissipation monitoring (QCM-D). Fluorescence and QCM-D both confirmed strong reduction of protein adsorption when compared to plasma-polymerized coatings and pristine poly(ethyleneterephthalate) (PET). Moreover, almost no platelet adhesion was observed after 15 min perfusion in whole blood. Altogether, our results suggest that primary amine-rich plasma-polymerized coatings offer a promising stable and versatile method for PEG grafting in order to create nonfouling and nonthrombogenic surfaces and micropatterns.

## 1. Introduction

Minimizing nonspecific interactions occurring between surfaces and biological species (e.g., proteins and cells) is of paramount importance in many devices including microfluidic, diagnostic, and implantable vascular devices. Indeed, the performance of small-diameter vascular grafts (<6 mm) made of poly(ethyleneterephthalate) (PET) or poly(tetrafluoroethylene) (PTFE) has been demonstrated to be drastically restricted by thrombotic occlusion, which is initiated by protein and platelet interactions with the graft surface [1]. Surface modification, by incorporation of hydrophilic polymers such as polyethylene glycol (PEG), has been shown to reduce nonspecific protein adsorption [1, 2]. PEG presents several advantages since it is a water soluble, synthetic, nonimmunogenic [3], and nontoxic [4] polymer approved by the FDA for internal consumption [5]. Furthermore, PEG coatings have been reported to exhibit low degree of protein adsorption [2] and platelet or cell adhesion [6]. Finally, PEG end-groups can also be used to graft biomolecules harboring desirable activities [7].

Several strategies have been proposed for PEG immobilization on biomaterial surfaces, including simple direct adsorption [8], radiation and chemical cross-linking approaches [9], and self-assembled monolayers [10]. In most cases, these approaches were shown to improve repellence of proteins and platelets *in vitro*, but results were variable and PEG coating sometimes failed to perform equally well *in vivo* [11], most likely due to insufficient stability of the PEG coating [12]. These results strongly suggest that the grafting method is an important design criterion in order to achieve both coating stability and performance. While simple adsorption is flexible and convenient, its efficacy is limited by the tendency of PEG to elute off the surface [13]. Stable PEG coatings generated by direct covalent chemical coupling to substrates have already been reported [14]. However, this approach is far from being versatile since it relies on the availability of compatible functional groups on both PEG and the host surface as well as on their respective surface densities.

In addition to the grafting method, the type of PEG molecule and its density after grafting play key roles in the prevention of protein adsorption: resistance to protein adsorption mainly depends on PEG chain length, grafting density, hydration, surface charge and conformation [15]. In this regard, star-shaped or multiarm PEGs are advantageous because of their molecular architecture and long chain length, which enable higher grafting density than with linear PEG [7, 16, 17]. Additionally, star PEG offers high density of functional groups that allow subsequent grafting of selected biomolecules designed to further tailor surface properties [7, 17]. Here, we present a novel method for grafting stable star PEG, which can be applied to a large variety of biomaterials (polymers, ceramics, metals and semiconductors used in biomedical applications); it also enables one to create various deposit geometries such as micro-patterns. To achieve this goal, we took advantage of stable primary amine-rich plasma-polymerized thin film coatings, developed and characterized previously in our laboratories as reported in [18–20]. More specifically, a low-pressure plasma-polymerized coating prepared from a mixture of ethylene and ammonia (hereafter “LP”), with high concentrations of nitrogen ([N] = 16%) and primary amines ([NH_2_] = 7.5) [18, 20], has been used. In the present work, the ability of this coating, combined with star PEG to create protein and platelet-repellent surfaces, has been studied. Covalent coupling of star PEG was first investigated on amino-coated glass substrates to optimize the method, as assessed by static contact angle and XPS analysis. Next, PEG coatings were created on LP-coated quartz crystals for protein adsorption studies by quartz crystal microbalance with dissipation monitoring (QCM-D). Finally, the ability of PEG coatings to decrease protein adsorption and platelet adhesion on PET films was confirmed by fluorescence microscopy and an *in vitro* perfusion platelet adhesion assays, respectively.

## 2. Materials and Methods

### 2.1. Chemicals and Reagents

Amino-coated glass slides (10 × 10 mm^2^) were purchased from Erie Scientific Co. (Portsmouth, NH, USA), and 50 *μ*m-thick poly(ethyleneterephthalate) (PET) film was purchased from Goodfellow (Huntingdon, England). Albumin Texas Red conjugate, Fibrinogen, PBS (pH 7.4), prostacyclin (PGI_1_), and Hanks' Balanced Salt Solution (HBSS) were obtained from Sigma Aldrich Canada Ltd. (Oakville, ON, Canada). A 4-arm PEG with N-hydroxy succinimide (NHS) terminal functional groups (PEG-NHS; MW = 10 kDa) was purchased from Creative PEG Works Inc. (Winston Salem, NC, USA). Parallel bar grids 3.05 mm in diameter were purchased from Electron Microscopy Sciences (Hatfield, PA, USA). 

### 2.2. Coating Preparation

#### 2.2.1. Plasma Polymerization

Plasma-polymerized ethylene coatings containing bonded nitrogen (LP) were deposited on PET and on gold-plated QCM-D crystal surfaces using a low-pressure radio-frequency (R.F.) glow-discharge plasma reactor, as extensively described elsewhere [18]. Briefly, a mixture of anhydrous ammonia (NH_3_) and ethylene (C_2_H_4_) (of 99.9% and 99.5% purity, resp., Air Liquide Canada Ltd., Montreal, QC) was admitted into a cylindrical aluminum/steel reactor chamber at flow rates of 15 and 20 standard cubic centimeters per minute (sccm), respectively. This gas ratio (*R* = 0.75) was based on a recent study that revealed high concentrations of nitrogen ([N] = 16%) and primary amines ([NH_2_] = 7.5%) a smooth surface, and good stability in air and in aqueous solvents [20] in the resulting coating, along with very low solubility. The low-power (*P* = 10 W) plasma was created at a pressure of 80 Pa, resulting in a negative dc bias voltage of −40 V. The duration of deposition, 10 min, led to 80–90 nm thick LP coatings.

#### 2.2.2. Grafting of Star PEG

**Figure 1:**
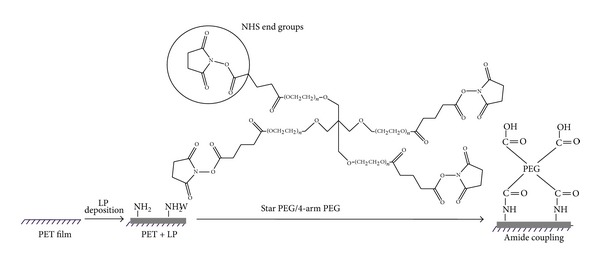
Schematic diagram of star PEG covalent binding reaction. Either one or two N-hydroxyl succinamide (NHS) terminal groups of star PEG react directly with primary amines through ester-amine reactions to form stable amide bonds; the remaining terminal groups do not participate in coupling, due to steric constraints [22] and hydrolyze to carboxylic acid groups during the reaction.

The PEG grafting procedure was performed on both amino-coated glass slides and on LP-coated surfaces. Of course, both contained primary amines with which the N-hydroxy succinimide (NHS) terminal groups on PEG readily react under slightly basic conditions (pH = 8.5) to form stable amide bonds [21], as shown in Figure 1. Prior to PEG grafting, the aminated glass slides were cleaned, in order to remove organic contaminants, using chloroform (99% purity, Fisher Scientific) for 2 min, followed by rinsing twice with Milli-Q water in an ultrasonic bath and drying with a flow of compressed nitrogen. PEG-NHS solutions at different concentrations (0.55, 1.66, 5, and 15% w/v) were prepared by dissolving in 0.025 M phosphate buffer (0.025 M NaH_2_PO_4_ + 0.025 M Na_2_HPO_4_, with drops of 0.01 M NaOH to adjust to pH 8.5). In order to prevent hydrolysis of NHS terminal groups, the PEG solution was immediately deposited on aminated surfaces for 2 h at room temperature. The PEG solution was then removed, and the slides were rinsed for 2 min with PBS, followed by Milli-Q water (2 times) in an ultrasonic bath. Finally, the slides were dried with nitrogen gas stream.

### 2.3. Surface Characterization

#### 2.3.1. X-Ray Photoelectron Spectroscopy (XPS)

The chemical composition of PEG-modified surfaces was characterized by XPS using a VG ESCALAB 3MkII instrument with nonmonochromatic Mg K*α* radiation. To minimize the effect of the substrate, spectra were acquired at a 70° take-off angle. For each sample, survey (0–1200 eV) and high-resolution spectra for C1s and O1s were recorded at pass energy of 100 eV and 20 eV, respectively. Charging was corrected by referencing all peaks with respect to the carbon (C1s) C–C, C–H peak at 285.0 eV. Advantage v4.12 software (Thermo Electron Corporation, Waltham) was used to quantify the constituent elements after Shirley-type background subtraction, by integrating the areas under relevant peaks.

#### 2.3.2. Static Water Contact Angle

The wettability of the aminated glass and LP-deposited surfaces before and after PEG grafting was assessed by static water contact angle measurements, using a Ramé-Hart Inc., Model 100–00 115 goniometer. Three measurements were taken on each surface using Milli-Q water as probe liquid (2 *μ*L drop size), and the experiments were repeated on three independent samples.

### 2.4. Protein Adsorption Studies

#### 2.4.1. QCM-D

QCM-D (Model Q-sense E4, Q-Sense AB, Sweden) was used to monitor fibrinogen adsorption on LP and star PEG-grafted surfaces. This technique has been recognized as a sensitive tool to investigate protein adsorption since it allows real-time followup of mass and viscoelastic properties of the adsorbed layer by measuring the changes in frequency and energy dissipation of an oscillating quartz crystal [23]. Gold-quartz crystals (5 MHz, Q-sense AB, Sweden) were coated with LP followed or not by star PEG grafting using PEG solutions of various concentrations (0.55, 1.66, 5, and 15% w/v) as described above. QCM-D assay was performed at 37°C using human fibrinogen (340 kDa) solution (0.5 mg/mL in PBS). Crystals were first exposed to PBS with a flow rate of 50 *μ*L/min until a stable baseline was reached for frequency and dissipation; a fibrinogen solution was then flowed for 2 h followed by rinsing with PBS for 30 min. Protein adsorption on 4 different surfaces was compared each time by following changes in frequency (Δ*f*) and dissipation (Δ*D*), and each surface was tested at least three times.

Since adsorbed protein forms a viscoelastic film, the Sauerbrey relation overestimates the real mass of adsorbed proteins onto the surface [24]. The Voigt viscoelastic modeling in QTools (Q tools, Q-sense AB, Sweden) was thus applied to estimate the adsorbed mass per surface unit by considering both frequency (Δ*f*) and dissipation (Δ*D*) shifts [24]. All the overtones were used except the first one to normalize the data and to estimate the adsorbed mass of protein. It is important to note that the estimated mass included protein molecules in addition to water trapped in the layer. The following parameters were used for modeling the data as described by Weber et al. [25]. The layer density was fixed at 1200 kg/m^3^. Parameters fitted were (i) layer viscosity between 0.001 and 0.01 kg/ms and (ii) layer thickness between 10^−10^ and 10^−7^ m. 

The stability of the coating was assessed by incubating 5% PEG-grafted crystals in PBS(37°C, pH 7.4, 5% CO_2_) up to 4 weeks. Samples were then rinsed with Milli-Q water and dried with nitrogen gas. QCM-D assay was performed as described previously to compare fibrinogen adsorption on LP (*n* = 1) with that on 5% PEG was immersed for 1 day (*n* = 1), or for 4 weeks in PBS (*n* = 2). The experiment was performed in triplicate. Between each experiment, gold crystals were extensively cleaned to remove LP and PEG for subsequent experiment. Crystals were cleaned by incubating them for 15 min in a solution containing Milli-Q water, ammonia, and hydrogen peroxide (ratio of 5 : 1 : 1, 75°C), followed by extensive rinsing with Milli-Q water. Then they were dried with N_2_ gas stream and exposed to UV and ozone for 10 min in a UV/ozone ProCleaner (Bioforce Nanosciences, Inc. Model ProCleaner 110), as reported in [26].

#### 2.4.2. Fluorescence Measurements

To confirm these results on more realistic surfaces and, simultaneously, confirm the non-fouling properties with a smaller protein size, albumin (66 kDa) adsorption on bare PET, LP- and LP-PEG-coated PET was studied using fluorescence microscopy. The samples were immersed into a solution of Albumin Texas Red conjugate (0.2 mg/mL in PBS) for 2 h at room temperature (surfaces being protected from light exposure). Surfaces were then washed thoroughly with PBS to remove unbound protein, dried with a stream of nitrogen and examined under a fluorescence microscope (Nikon Eclipse E600 mounted with a Photometrics CoolSnap HQ2 CCD camera) at 10x magnification. Fluorescence intensity (fluorescence excitation and emission of 596 and 615 nm, resp.), which directly correlated with the amount of albumin adsorbed on the surface [27], was measured using the NIS-Elements AR (version 3.0) Nikon software, and the background was subtracted for each sample. Intensity for fluorescence measurements is given as counts per second (cps). 

#### 2.4.3. Micro Patterning

The ability to generate nonfouling microscale patterns on PET substrate was investigated using LP deposition. Micro-patterns were generated by placing electron microscopy grids (consisting of 184 *μ*m wide parallel bars separated by 92 *μ*m) over PET substrates before LP deposition. Then, PEG grafting was performed by covalent binding, and unbound PEG was removed by rinsing with PBS in an ultrasonic bath as explained above. Samples were then immersed in Albumin Texas Red conjugate (0.2 mg/mL in PBS) for 2 h at room temperature, and surfaces were washed thoroughly with PBS to remove unbound protein. Finally, substrates were examined by fluorescence microscopy.

### 2.5. Platelet Adhesion

#### 2.5.1. Platelet Isolation and Labeling

Platelet adhesion on control and modified surfaces was evaluated by perfusion tests using radiolabeled platelet in fresh human blood. This part of the study has been approved by the human ethical committee of the Montreal Heart Institute. All subjects gave informed consent and were free from drugs interfering with platelet function for at least 2 weeks before blood sampling. Platelet isolation and labeling as well as the perfusion experiments were conducted as described previously [28]. A 60 mL sample of venous blood from each subject was anticoagulated with 6 mL of D-Phenylalanyl-L-prolyl-L-arginine chloromethyl ketone (PPACK) in saline (50 nM final concentration, Calbiochem, QC, Canada), and a 30 mL with anticoagulant citrate dextrose (ACD, Baxter, Mississauga, Canada). The ACD blood was used to isolate and radiolabel platelets. Briefly, platelet-rich plasma (PRP) was isolated by centrifugation for 15 min at 1800 rpm. PRP was then centrifuged for 10 min at 2200 rpm to separate platelets from platelet-poor plasma (PPP). The pellet of platelets was suspended with Hanks' Balanced Salt Solution (HBSS) containing 0.5 *μ*g/*μ*L of prostaglandin E1 (PGE_1_) and centrifuged for 10 min at 2200 rpm. The platelet pellet was resuspended with 2 mL of HBSS containing PGE_1_ and incubated with 250 *μ*Ci of Indium^111^ Oxine (GE Healthcare Canada Inc., Burlington, Ontario) for 15 min at room temperature, followed by centrifugation for 8 min at 2100 rpm. The supernatant was removed and the radiolabeled platelet pellet resuspended in 10 mL of PPP and finally mixed with the 60 mL of the PPACK blood. Platelet count of each sample was measured using Coulter Act Diff cell counter (Beckman Coulter, Canada). For reference, 10 *μ*L of blood containing labeled platelets was measured using a gamma counter (Minaxi 5000, Packard Instruments).

#### 2.5.2. Platelet Adhesion

Platelet adhesion was conducted using plexiglas perfusion chambers that mimic the tube-like cylindrical shape of blood vessels, as described previously [28, 29]. The samples (bare PET, LP-coated PET, and PEG-coated PET prepared with 5% w/v PEG were placed in the perfusion chambers containing a window of 2 mm internal diameter ×  10 mm long, permitting direct exposure of the samples to the blood. The connection between the chambers and the peristaltic pump was performed using nontoxic and nonpyrogenic Tygon flexible surgical tubes (Tygon R-100, Fisher Scientific, Canada). A thermostatically controlled water bath was used to maintain the perfusion system at 37°C. The samples within the perfusion chambers were directly exposed to the blood containing ^111^in labeled platelets for 15 min at a flow rate of 40 mL/min and a shear rate of 853 sec^−1^, followed by rinsing with a buffered formalin solution for fixation. At the end of each experiment, the radioactivity on the exposed surfaces was measured using a gamma counter (Minaxi 5000, Packard Instruments). Platelet adhesion on each test surface was calculated from the known radioactivity of reference and platelet count in the blood in count per minute (cpm), using the following equation:

(1) Platelet adhesion=(In⁡111 cpm in exposed segment)×(No. of platelets/mL blood)/In⁡111 cpm/mL bloodexposed surface (cm2).

The experiment was repeated for each test surface using three different healthy blood donors. Results were also compared to fresh endothelium and injured arterial surfaces.

#### 2.5.3. Scanning Electron Microscopy

After platelet adhesion assay, each surface was observed by scanning electron microscopy (SEM) to assess platelet adhesion and morphology. The specimens were dehydrated through a series of graded ethanol solutions (30%, 50%, 70%, 95%, and 2x100% v/v) and subjected to CO_2_ critical point drying (E3000, Polaron, Quorum Technologies). The dried specimens were finally sputter-coated with gold and observed under SEM using a Hitachi S-3600N (Hitachi High-Technologies, Canada).

### 2.6. Statistical Analysis

All the results were expressed as mean ± standard deviation. Statistical analysis was carried out using one-way ANOVA analysis followed by Tukey's post hoc test. Student's *t*-test was used when comparing two groups. *P* < 0.05 was considered to be statistically significant.

## 3. Results

### 3.1. Surface Characterization

**Figure 2:**
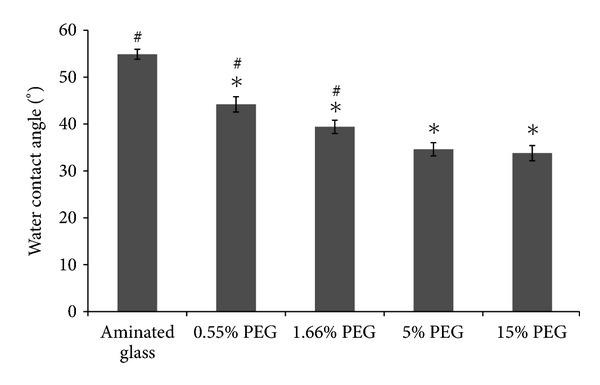
Static water contact angles on unmodified aminated glass and after PEG grafting using PEG solution at concentrations of 0.55, 1.66, 5, and 15%w/v. Results are expressed as mean ± SD, *n* = 3. *Significantly different from aminated glass (*P* < 0.001) ^#^Significantly different from 5% PEG (*P* < 0.001).

In order to evaluate the efficiency of our PEGylation protocol, water contact angles were measured on aminated glass before and after PEG (Figure 2). While aminated glass exhibited a relatively hydrophilic surface (54.9 ± 1.1°), the contact angle significantly decreased after star PEG grafting as a function of its coupling concentration. As can be seen in Figure 2, no significant difference was observed between 5 and 15%; very similar results were obtained for PEG grafting on PET films after LP deposition (data not shown). LP coatings, on their own, exhibited a contact angle of about 56.1 ± 0.6°.

**Figure 3:**
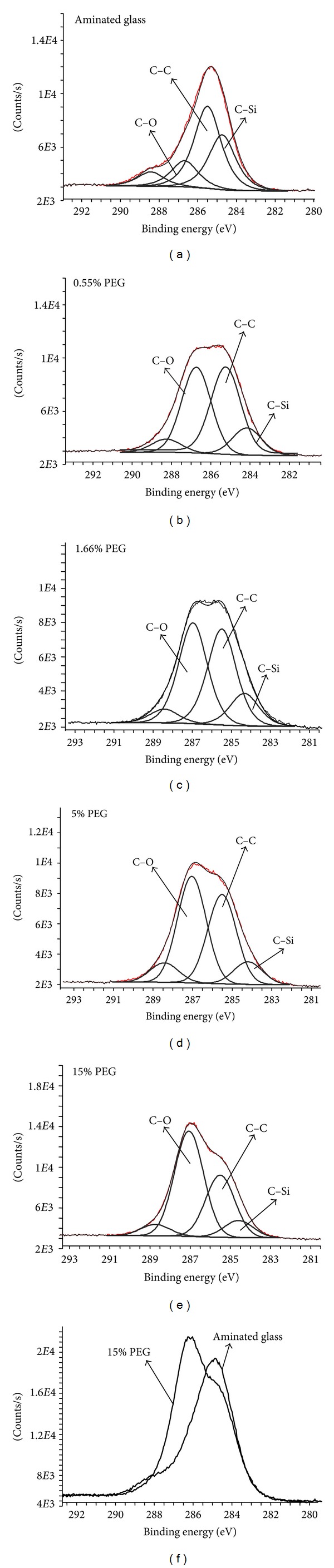
XPS high-resolution C1s scans of aminated glass (a) before and (b-e) after star PEG grafting at various coupling concentrations (0.55, 1.66, 5, and 15% w/v). Note that the relative intensity of the C–O peak increased with the PEG coupling concentration; (f) Overlay spectra of 15% PEG-modified on unmodified aminated surface.

XPS measurements further confirmed star PEG presence after the covalent coupling procedure on aminated glass surfaces. Survey scans were used to study changes in elemental composition of modified and unmodified substrates.  Table 1 shows the decrease of silicon (Si) and Nitrogen (N) concentration, in parallel to the increase in carbon (C) on all PEG-modified surfaces, indicating the presence of PEG. These results were further confirmed by high-resolution C1s and O1s scans. As shown in Figure 3(a), four different peaks were identified on the aminated glass control; the main two peaks (binding energies of 285 eV and 284 eV) corresponded to carbon-carbon (C–C) and carbon-silicon (C–Si) bonding, respectively. The low-intensity peak around 286.3 eV was attributed to a mixture of C–N and C–O bonds, for which binding energies are too close to be discriminated. This peak most likely came from the amination process used by the manufacturer, since commercial aminated glass substrates are generally produced by amino silylation using amino propyl triethoxy/methoxy silane. Therefore, it is probable that this peak combine C–O (from triethoxy) and C–N (from C–NH_2_ end groups) groups. The other small intensity peak at higher energy level (288.4 eV) was attributed to the presence of carboxyl groups resulting from surface contamination [16]. High-resolution scans of all-star PEG-grafted surfaces presented a large increase of the peak around 286.5 eV, in agreement with the presence of C–O bond in PEG. As can be seen in Figures 3(b)–3(e), the relative intensity of this peak increased with star PEG coupling concentration, hence highly suggesting that the density and/or thickness of PEG coating increased with PEG concentration. XPS analysis was also performed on LP surfaces. However, due to the complexity of LP composition, in particular the presence of C–N bond, whose binding energy is close to that of C–O groups in PEG [30], we were unable to conclude about C–O increase on these substrates.

### 3.2. Protein Adsorption Studies

**Figure 4:**
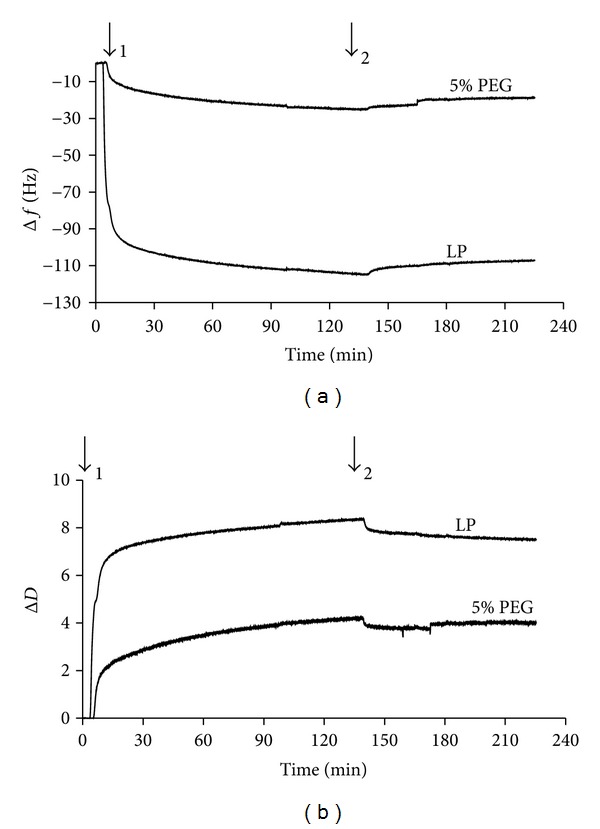
QCM-D real-time change in resonance frequency (Δ*f*) and dissipation (Δ*D*) related to modified (5% PEG) and unmodified LP surfaces upon exposure to fibrinogen solution (0.5 mg/mL; (1)) followed by rinsing with PBS (2).

**Figure 5:**
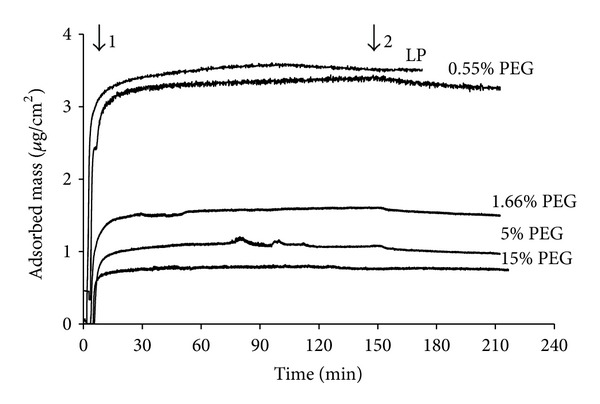
Time-resolved effect of various PEG coupling concentrations on fibrinogen (0.5 mg/mL) adsorption. QCMD data were analyzed according to the Voigt model. Time of fibrinogen injection (1) and rinsing with PBS (2) are indicated.

**Figure 6:**
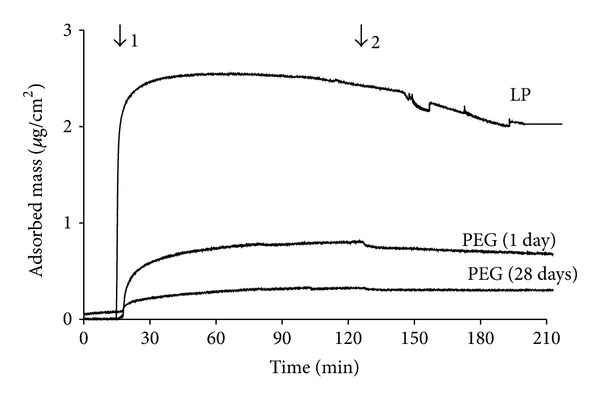
Fibrinogen (0.5 mg/mL) adsorption on LP and on PEG-modified surfaces immersed in PBS over a period of 1 or 28 days. QCMD data were analyzed according to the Voigt model. Time of fibrinogen injection (1) and rinsing with PBS (2) are indicated.

The non-fouling properties of our LP-PEG coatings were first investigated by monitoring fibrinogen adsorption using QCM-D.  Figure 4 presents an example of change in resonance frequency (Δ*f*) and dissipation (Δ*D*) when LP- and PEG- (5%) coated Quartz crystals were exposed to a solution of fibrinogen (0.5 mg/mL). On LP surfaces, introduction of the fibrinogen solution led to rapid decrease of the resonance frequency, indicating fibrinogen adsorption, while the related increase in dissipation indicated that the adsorbed protein layer was viscous. A subsequent rinse with PBS (pH 7.4) induced only slight changes, indicating that only a small amount of bound fibrinogen was dissociated when rinsing with buffer, and most of the protein was irreversibly adsorbed [23]. Δ*f* and ΔD were much reduced on PEG-modified surface. Typical mass variation over time with the various PEG concentrations is presented in Figure 5. The reduction in fibrinogen adsorption resulting from the various PEGylation treatments, compared to pristine LP surfaces are summarized in Table 2. Fibrinogen adsorption decreased with rising PEG concentration, reaching a maximum reduction of 79 ± 11% for 5% w/v PEG solution. Increasing PEG concentration from 5% to 15% did not result in a significant difference in subsequent fibrinogen adsorption (*P* = 0.78). Finally, after 4 weeks of immersion in PBS, 5% PEG-coated surface still exhibited strong reduction of fibrinogen adsorption (89 ± 7%) compared to LP (Table 2 and Figure 6).

**Figure 7:**
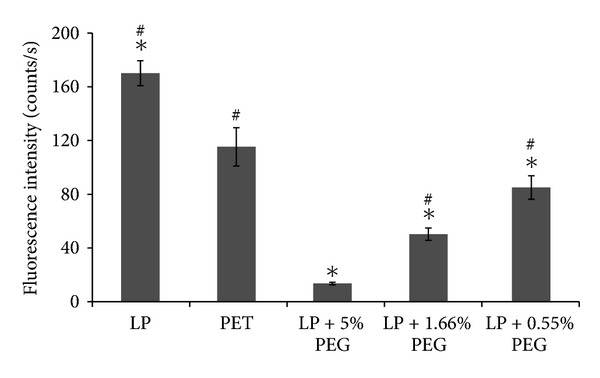
Fluorescence detection of adsorbed Texas Red labeled albumin (0.2 mg/mL) on bare PET, LP alone, and LP-PEG-coated PET. Results are expressed as mean ± SD (*n* = 4). Background was subtracted from each surface. *Significantly different from PET (*P* < 0.001), ^#^Significantly different from LP+ 5% PEG (*P* < 0.001).

In parallel, Texas Red conjugated albumin (66 kDa, 0.2 mg/mL) adsorption in static condition was also investigated using fluorescence microscopy in order to allow comparison with PET surfaces (Figure 7). Since no significant difference had been observed by QCM-D between 5% and 15% PEG conditions, only coatings obtained with in-solution concentrations of PEG up to 5% were compared to LP and to bare PET surfaces. While LP coating increased albumin adsorption compared to bare PET films, further PEG grafting strongly decreased protein adsorption. PEG coatings were even effective at low in-solution PEG concentrations,d and the amount of albumin adsorption decreased as PEG coupling concentrations were increased. Of interest, all PEG concentrations enabled to decrease albumin adsorption below PET control (*P* < 0.001). On 5% PEG, fluorescence was decreased by 92% and 88% compared to LP and PET, respectively.

**Figure 8:**
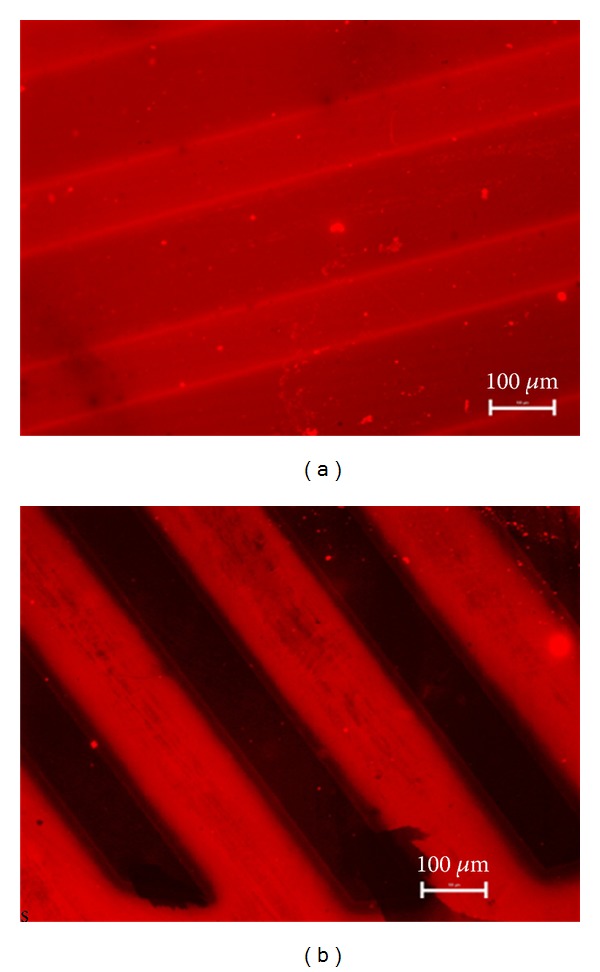
Fluorescence microscopy images of LP micro-patterns on PET surfaces after exposure to albumin Texas Red conjugate. Parallel pattern surface (a) without PEG grafting and (b) modified with PEG.

Texas Red conjugated albumin was also used to demonstrate the applicability of our LP deposition method to the generation non-fouling micro-patterns (Figure 8). While only slight difference was observed between PET and PET+LP coated regions (Figure 8(a)), subsequent PEG grafting led to non-fouling areas clearly distinct from bare PET regions that manifested strong adsorption (Figure 8(b)). 

### 3.3. Platelet Adhesion

**Figure 9:**
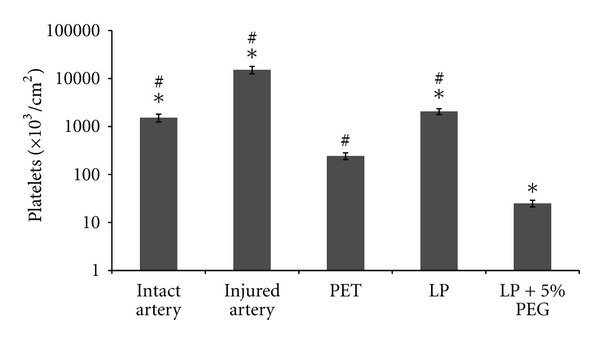
Platelet adhesion on an intact artery, injured artery, PET film, LP and 5%PEG- modified substrates. Results are expressed on a logarithmic scale, as mean ± SD (*n* = 4). *Significantly different from PET (*P* < 0.001) ^#^Significantly different from LP + 5% PEG (*P* < 0.001).

**Figure 10:**
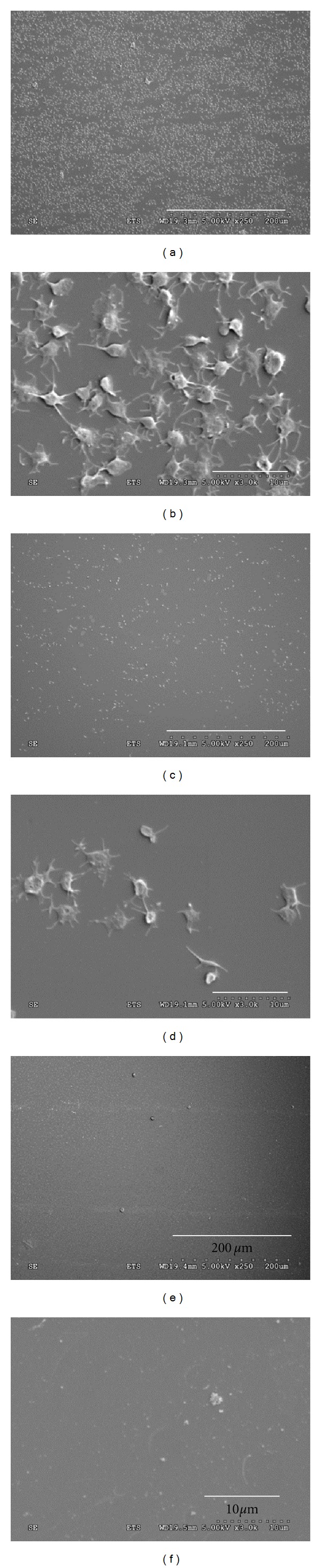
SEM visualization of platelet adhesion on LP (a, b), PET (c, d), and LP + 5% PEG-c (e, f) grafted surfaces.

The levels of platelet adhesion on the various surfaces after 15 minutes exposure to whole blood under perfusion are presented on Figure 9. As expected, LP coating on PET significantly increased platelet adhesion when compared to pristine PET (2060 × 10^3^ versus 244 × 10^3^ platelets/cm^2^); these levels were, however, much lower than those corresponding to injured arterial tissues (15,195 × 10^3^ platelets/cm^2^). Of interest, PEG grafting (5% PEG solution) on LP drastically decreased LP surface thrombogenicity, reaching levels about 10 times lower than those determined for bare PET control surfaces (25 × 10^3^ versus 244 × 10^3^ platelets/cm^2^; *P* < 0.001). Direct observation of each individual surface using SEM (Figure 10) confirmed that platelet adhesion was almost abolished on PEG surfaces, since no platelets were observed on these surfaces. In contrast, relatively high level of platelet adhesion was noticed after LP treatment and platelet morphology indicated that some of them were activated. Finally, although only few platelets were identified on PET surfaces, their morphology corresponded to elongated pseudopodia and directed towards platelet activation [31], which has been reported to contribute to the recruitment of other platelets and blood cells such as leukocytes during long-term contact with the blood.

## 4. Discussion

The development of a versatile method for star PEG coating is of great interest to generate nonfouling surfaces for several applications; these include cardiovascular implants and microdevices directed towards biomedical and analytical applications, among others. To be effective, a dense, uniform, and stable PEG coating must be created at these surfaces [13, 16, 22]. In this study, we took advantage of high primary amine content ([NH_2_] = 7.5%) of LP coatings [20] for subsequent covalent grafting of NHS-PEG. Our amine-rich coating was combined with star-shaped PEG because of the latter's molecular architecture that offers high surface coverage and high steric hindrance towards protein adsorption [17]. Indeed, for similar chain lengths and molecular weights, star-shaped polymer brush variants have been demonstrated to possess higher density and greater steric repulsive forces against adsorbing proteins when compared to linear PEGs [32]. It has also been demonstrated that protein resistance can be enhanced with PEG molecules comprising a large number of hydrogen bond donors, such as carboxylic acid and hydroxyl functional groups [33]. In this study, we have successfully grafted star PEG in a covalent fashion onto aminated surfaces, as indicated by contact angle measurements and by XPS. However, the presence of surface-adsorbed PEG cannot be completely excluded, though unlikely because rinsing in the ultrasonic bath most likely removed loosely bound PEG from the surface.

Increasing PEG concentration during coupling greatly increased the density of the final coating, up to 15% PEG. While contact angle measurements showed no difference between 5 and 15% PEG, the relative intensity of the C–O peak was higher for 15% PEG. This suggests a higher density of the PEG coating that may in turn reduce protein adsorption, especially for small proteins such as albumin. However, owing to the high cost of star-shaped PEG and the modest improvement according to QCM-D assay when increasing concentration from 5 to 15%, we chose to evaluate the 5% PEG coating only, assuming that it would sufficiently reduce protein and platelet adhesion. Fibrinogen and albumin were chosen for protein adsorption studies due to the fact that they are two major constituents of blood plasma. Fibrinogen (340 kDa), in particular, plays a key role in platelet adhesion and aggregation [1]. Albumin, a smaller protein (66 kDa), was also tested. 5% PEG coating strongly decreased protein adsorption compared to LP surfaces, and fibrinogen adsorption was reduced by 79% and albumin by 92%, as deduced from QCM-D and fluorescence measurements, respectively. The slight discrepancy between QCM-D and fluorescent labeling results may be attributable to an overestimation of wet mass adsorption by QCM-D, in addition to inherent differences between dynamic (QCM-D) and static (fluorescence) assays for protein adsorption. Indeed, it has been already reported that QCM-D leads to 1.4- to 4-fold overestimation of adsorbed mass when compared to labeling or optical methods, depending on the type of protein [34]. However, protein adsorption could not be completely abrogated, even at high PEG coupling concentration. This result is most likely due to protein adsorption occurring at open spaces between the star PEG molecules [16], a process that can be favored by protein conformation changes initiated by steric constraints [35]. Studies have also shown that protein adsorption decreases as PEG chain length and density increase [7, 13, 36]. Hence, increasing PEG molecular weight and concentration may further decrease or completely inhibit protein adsorption with our amine-rich deposited polymer. 

Although it is difficult to compare our data with adsorption results published by various research teams since the amount of adsorbed protein depends on its concentration and on the measurement method, our coating generally compares well with those reported in the literature. Indeed, Unsworth and colleagues reported an 80% reduction of fibrinogen adsorption on surface with high PEG chain density [37], where PEG had been grafted on gold surface through chemisorption method. The reduction of albumin adsorption assessed by fluorescence here (>90%) is comparable to the results obtained by covalent coupling of PEG on silicone surfaces [38]. Other teams, however, showed that even more complete protein repellency can be achieved with PEG-containing copolymers (bulk modification). Weber and coworkers thus reported 95% reduction of fibrinogen adsorption on poly(DTEcarbonate) PEGylated copolymers (at 15 mol%) [25], while Zhou et al. [39] reported negligible fibrinogen adsorption on a polyurethane copolymer when PEG content was increased to 52.5%. However, these particular cases are very different from the method that we propose here, since the latter is much more versatile and can be implemented on any type of surface without modifying the bulk properties of biomaterials. Another interesting feature of the present method is its use to create fouling/non-fouling micro-patterns, as shown by our preliminary experiments with electron microscopy grids (Figure 8). Finally, the present PEG grafting method was demonstrated to exhibit excellent stability, as evidenced by strong reduction of fibrinogen adsorption over periods close to one month. Interestingly, PEG was found to be even more protein-resistant after 4 weeks of incubation in PBS than after 1 day in PBS. The reason for this observation is unclear, and further investigation would be needed using complementary surface analysis techniques and different incubation times to better assess PEG stability and conformation on the surfaces.

The low level of fibrinogen adsorption on PEG surfaces can be directly related to their ability to prevent platelet adhesion [6]. This was confirmed with our perfusion model using fresh human blood, which mimics physiological conditions. This test is believed to better estimate the non-thrombogenic potential of PEG-grafted surfaces than platelet adhesion tests performed under static condition [40], since the contact of platelets with a given surface is a dynamic process that involves adhesion, activation, secretion, and spreading. The relatively high level of platelet adhesion after LP treatment and their morphology by SEM indicate that LP attracts blood elements such as proteins and platelets. PEG grafting rendered the surface thromboresistant, as revealed by the negligible number of platelet adhesion determined in our platelet adhesion assay and further confirmed by SEM observations. The PEG surface also resists to cell adhesion and growth, as confirmed with Human Umbilical Vein Endothelial cell (HUVEC) (data unshown). 

The present PEG grafting method is versatile and is shown here to possess good stability in PBS, but how long PEG-coated implants would consistently exhibit protein resistance and prevent thrombosis *in vivo* needs further investigation since PEG long-term stability and efficiency to prevent thrombus formation *in vivo* is subject of controversy [41, 42]. Strategies combining non-fouling properties with the immobilization of antithrombogenic molecules, such as heparin, hirudin, or other direct thrombin inhibitors [42, 43] or of proadhesive peptides and growth factors to favor endothelialisation [44] should enable to solve this issue. The star PEG versatile grafting method developed here is compatible and promising for these strategies. Indeed PEG derivatives have been shown to be interesting molecular linker/spacer for bioactive molecules. Moreover, due to the steric constraints, either one or two terminal groups of star PEG are grafted to the surface, while the remaining groups do not participate in amide coupling and emanate outside of the surface. Hence, these terminal groups will be available for subsequent coupling of biomolecules.

## 5. Summary and Conclusion

A versatile method was optimized to create a non-fouling and non-thrombogenic coating that may be applicable to most biomaterial surfaces and enables micropatterning. The surfaces were not completely protein repellent and could be further optimized. However, platelet adhesion study suggests that star PEG-modified surfaces are non-thrombogenic. Moreover, the grafted PEG can also be utilized for further immobilization of bioactive molecules. In future steps, immobilization of growth factors, adhesion molecules, or antithrombogenic biomolecules to the PEG-grafted surface may be used to optimize its nonthrombogenicity, improve endothelialization, or favor healing around cardiovascular implants. 

## Figures and Tables

**Table 1:** Surface elemental concentration (in At.%) of C, O, Si, and N, as determined by XPS on aminated glass and PEG-modified surfaces using various star PEG coupling concentrations.

Surface	O%	C%	Si%	N%
Aminated glass	40.9	36.6	17.0	4.8
0.55% PEG	38.3	44.5	12.7	3.7
1.66% PEG	37.4	46.8	11.8	3.3
5% PEG	36.1	50.3	10.1	3.1
15% PEG	37.4	48.1	11.1	2.8

**Table 2:** Percentage of reduction of fibrinogen (0.5 mg/mL) adsorption compared to LP surface for different coupling concentrations of star PEG. Results are expressed as mean ± SD, *n* = 4.

Surface	Reduction of fibrinogen adsorption (%)
15% PEG	76 ± 18
5% PEG	79 ± 11
1.66% PEG	64 ± 11
0.5% PEG	34*

5% PEG after 4 weeks in PBS	89 ± 7%

*Result expressed as a mean; *n* = 2 only.
